# Control of Daily Transcript Oscillations in *Drosophila* by Light and the Circadian Clock

**DOI:** 10.1371/journal.pgen.0020039

**Published:** 2006-03-24

**Authors:** Herman Wijnen, Felix Naef, Catharine Boothroyd, Adam Claridge-Chang, Michael W Young

**Affiliations:** 1 Laboratory of Genetics, The Rockefeller University, New York, New York, United States of America; 2 Department of Biology, University of Virginia, Charlottesville, Virginia, United States of America; 3 Laboratory of Mathematical Physics, The Rockefeller University, New York, New York, United States of America; 4 Swiss Institute for Experimental Cancer Research (ISREC), Ecole Polytechnique Federale de Lausanne (EPFL), Lausanne, Switzerland; Princeton University, United States of America

## Abstract

The transcriptional circuits of circadian clocks control physiological and behavioral rhythms. Light may affect such overt rhythms in two ways: (1) by entraining the clock circuits and (2) via clock-independent molecular pathways. In this study we examine the relationship between autonomous transcript oscillations and light-driven transcript responses. Transcript profiles of wild-type and arrhythmic mutant *Drosophila* were recorded both in the presence of an environmental photocycle and in constant darkness. Systematic autonomous oscillations in the 12- to 48-h period range were detectable only in wild-type flies and occurred preferentially at the circadian period length. However, an extensive program of light-driven expression was confirmed in arrhythmic mutant flies. Many light-responsive transcripts are preferentially expressed in the compound eyes and the phospholipase C component of phototransduction, NORPA (no receptor potential), is required for their light-dependent regulation. Although there is evidence for the existence of multiple molecular clock circuits in cyanobacteria, protists, plants, and fungi, *Drosophila* appears to possess only one such system. The sustained photic expression responses identified here are partially coupled to the circadian clock and may reflect a mechanism for flies to modulate functions such as visual sensitivity and synaptic transmission in response to seasonal changes in photoperiod.

## Synopsis

Daily changes in sunlight dramatically affect the environment of animals like the fruit fly, a genetic model. To anticipate these changes, the fruit fly possesses a circadian molecular clock that regulates its behavioral activity and other physiology according to the time of day. The clock's mechanism is comprised of genes and their products (proteins and RNAs). One way the clock modulates physiology is by regulating other genes' RNAs. The signature of a circadian RNA is that its levels oscillate once each day. The present study uses microarrays and a novel statistical strategy to resolve how many genes are oscillating in the fly head and verify that there is only a single transcriptional clock. However, this study also finds a large number of genes that are directly regulated by the presence of light. These genes respond to light even in the absence of a functioning clock. Indeed, the light response depends on a signaling component in the retina that has an established role in vision. This represents a new mechanism by which an animal's physiology may be dynamically tuned to daily time.

## Introduction

The circadian clocks of cyanobacteria, protists, plants, fungi, and animals produce self-sustained physiological rhythmicity that resonates with daily fluctuations in the environment. Circadian clocks regulate the timing of diverse behavioral and physiological functions, and many of the molecular components of such clocks have been identified by genetic and biochemical studies. Feedback circuits regulated at the level of clock gene expression, clock protein accumulation, and clock protein degradation are thought to form most if not all circadian clocks. Interactions among these circuits appear to promote molecular oscillations of the clock components themselves and rhythmic gene activities that are subordinate to the clock. Entrainment of circadian clocks to environmental cycles of light and dark is best understood in *Drosophila* and *Neurospora*. In the former, the clock protein TIM (timeless) is rapidly degraded in response to light, causing resetting of the oscillating mechanism. In *Neurospora,* light induces expression of the clock gene *frequency,* which encodes the central transcriptional repressor in the *Neurospora* clockworks (for reviews, see [1–9]).

While current knowledge of the molecular clock circuits can account for the autonomous oscillator function and photic entrainment of the circadian clock, many questions regarding the connections between molecular oscillations and overt rhythms remain unanswered. For example, rhythmic synaptic release of the neuropeptide PDF (pigment dispersing factor) from a subset of pacemaker neurons in the adult brain is required for maintenance of circadian locomotor behavior under constant conditions [10] and for the increased locomotor activity that occurs in anticipation of dawn in the presence of an environmental photocycle [11,12], but it is unclear how PDF release is governed by the molecular clock circuits. One of the most obvious mechanisms for generating molecular output from the circadian clock is the induction of rhythmic transcription of output genes that do not feed back on the clock circuits, but instead contribute directly or indirectly to the generation of overt rhythms. The fact that functional clock circuits are widespread throughout the bodies of animals (for review, see [13,14]) suggests that tissue-specific physiological rhythms may be specified by locally generated circadian transcriptional profiles. To test the hypothesis that signals from both the pacemaker neurons and peripheral clock tissues may be initiated at the level of clock-controlled gene expression, we and others have conducted genome-wide surveys to characterize the extent and nature of rhythmic transcription in the fly head [15–19]. Along with similar studies in *Arabidopsis,* mouse, rat, and *Neurospora* [20–27], these studies established the idea that transcriptional modulation of mRNA levels is a widely used mechanism of clock output.

One surprising result from these studies was the poor overlap between the proposed circadian gene lists of the five different fly circadian microarray studies [15–19]. Despite the fact that a number of predicted circadian transcript oscillations were validated independently by Northern blot analysis or real-time PCR [15,16,19], this between-study discrepancy raised serious concerns regarding the true extent of the intrinsic clock-dependent expression program. To address this controversy, we developed a new analytical strategy that allowed us to measure intrinsic rhythmicity in time-series microarray data. When applied to wild-type data, this method revealed wide-spread 24-h rhythmicity in gene expression levels. A meta-analysis of all available data clearly resolved that circadian modulation affects many more mRNAs in the fly head than is indicated by the overlap of published transcript profiling studies.

Our new analytical approach also allowed us to address three other questions that are central to animal circadian biology: (1) the importance of ultradian or infradian periodicity in gene expression, (2) the existence of alternative circadian oscillators, and (3) the relationship between diurnal and circadian regulation of gene expression.

First, is a significant part of the transcriptome regulated by ultradian or infradian rhythms in the 12- to 48-h range? In wild-type data 24-h rhythms were selectively enriched in comparison to ultradian or infradian periods. Moreover, unlike circadian rhythms, expression rhythms at non-circadian period lengths such as 12 or 16 h were no more prevalent in real wild-type or mutant data than in randomly permuted data.

Second, is the known clock, which controls behavior and requires *tim* the only transcriptional clock in *Drosophila?* No preference for circadian rhythmicity was found in *tim* null-mutant *(tim^01^)* data, a result consistent with the hypothesis that this mutant lacks a functional circadian clock [28]. Absence of 24-h rhythmicity in *tim^01^* adult heads was further supported by a systematic appraisal of candidate gene profiles by Northern blotting. In addition to this loss of circadian gene activity, our studies failed to detect gene expression rhythms with alternative periods in the range of 12 to 48 h in *tim^01^* flies, indicating that there are no residual transcriptional clocks in these mutants. Our results strongly suggest that the extensive program of rhythmic control detected in wild-type flies reflects function of a single circadian molecular oscillator.

Third, we address the issue of how light affects daily transcript oscillations in the fly head. Does it do so solely through its effect on the clock, or are there other pathways of daily gene regulation? If there are other pathways, how do they interact with the clock-controlled modulation? Stratification of the data into entrainment and free-running days allowed us to search for mRNA expression profiles that vary periodically as a function of an imposed photocycle in both wild-type and *tim^01^* flies. Our method uncovered a set of transcripts that show sustained up- or down-regulation in response to light. These light responses are abrogated in flies mutant for the *norpA* (no receptor potential A) gene, which have a defect in visual transduction [29], but persist in the *cry^b^* (cryptochrome-baby mutant), which has a specific defect in light-responsiveness of the clock [30]. Independently selected groups of light-regulated and clock-regulated transcripts were found to overlap significantly. On the basis of the associated gene functions and spatial expression patterns, we believe the newly identified light-driven expression system is likely linked to control of vision and synaptic function.

## Results

### Systematic Enrichment for Circadian Transcript Regulation in Wild-Type Adult *Drosophila* Heads

**Figure 1:**
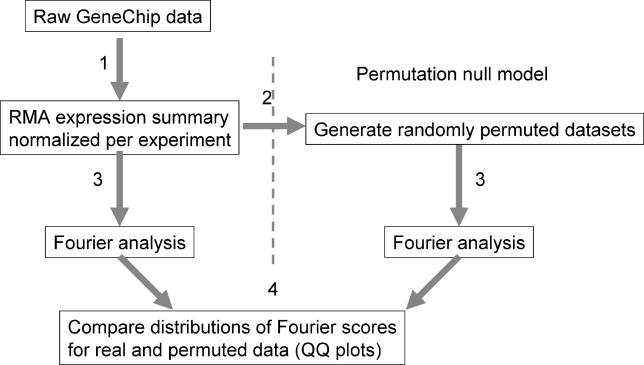
Strategy for Detecting Intrinsic Expression Rhythms (1) Expression values are calculated from raw data using the RMA signal algorithm. Data for each time-course experiment are mean-centered and standardized. (2) Next, 1,000 permuted datasets are derived by randomizing the order of time points within experiments. (3) Both the real and permuted data are subjected to Fourier analysis to determine of the strength of periodic expression components. (4) Finally, the distribution of periodic expression components in the real data and the permuted data are compared and illustrated by QQ plots.

**Figure 2:**
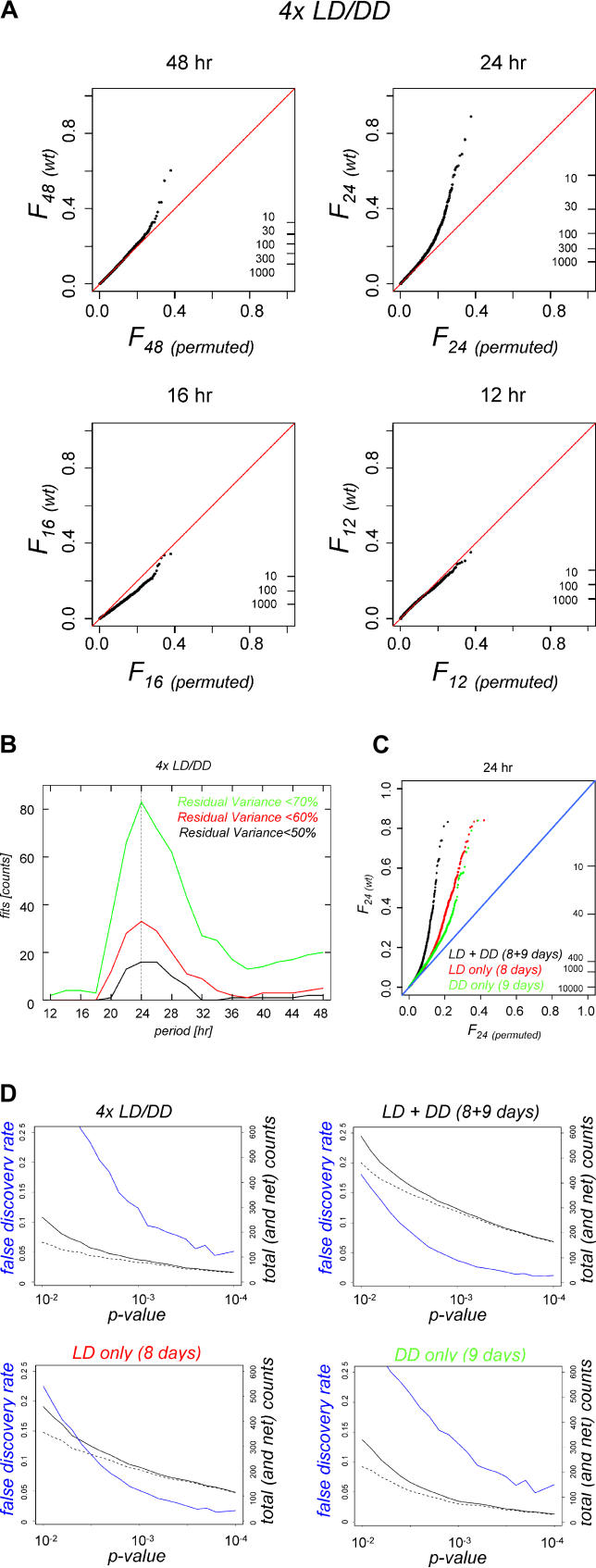
Genome-Wide Detection of Rhythmicity in Wild-Type LD/DD Time Series (A) QQ plots of spectral power (squared Fourier components) at four different periods are shown for microarray data collected from four wild-type 2-d LD/DD time course experiments. Upward deviations from the diagonals indicate global enrichment in time traces with that periodicity, while downward deviations indicate depletion. The ticks on the right show the number of profiles that exceed a certain Fourier value, emphasizing that the enrichments at 24 and 48 h are tail effects that affect no more than approximately 5% of the genome. Fourier components with 16 and 12-h periods do not show similar deviations. (B) Strongest oscillations in the 4x LD/DD wild-type data occur for periods near 24 h. The data were fitted with cosines, and then sets of transcript profiles were chosen based on how much of their variance was due to the cosine fit. Invariably, fitting the data with 24-h period cosines produced the largest sets (as indicated by the vertical axis). Each of the three curves is the same analysis done at different levels of fit stringency, with the black curve (“Residual Variance < 50%”) being the most stringent selection. (C) The contours of an extensive circadian expression program emerge from a comprehensive meta-analysis. The QQ plots (same format as for panel [A]) represent the 24-h spectral power distribution across all available wild-type LD and DD time-course data (this study; [15–18]; eight and nine independent days for, respectively, LD and DD). Analyses for LD data, DD data, and the combined LD + DD set are shown separately. (D) Reliable detection of extensive daily expression programs. The diagrams show the FDR (blue line), as well as the raw and background-corrected numbers of daily transcript oscillations (total and net counts; solid and dashed black lines) as a function of the selective *p*-value cut-off that is applied (see Materials and Methods). Compared to the original 4× LD/DD data analysis (upper left panel) integrative analysis of the expanded LD + DD (8 + 9 d) dataset (upper right panel) is clearly more powerful. Note that the relative enrichment of 24-h periodic patterns is enhanced in the combined LD + DD data (upper right panel) set relative to the LD-only and DD-only subsets (lower left and right panels), and that substantially more daily oscillations are detected in LD versus DD conditions.

To identify high-confidence rhythmic gene expression, we compared the distribution of oscillatory statistics in wild-type microarray data with a permutation null-model as outlined in Figure 1. In such models the original time ordering is randomly scrambled to construct the distribution of scores expected for arrhythmic data. The results are illustrated by the quantile–quantile (QQ) plots in Figure 2A. In the QQ plots, the Fourier scores of real data (on the *y*-axis) are graphed against quantile Fourier scores associated with our null hypothesis (on the *x*-axis). Enrichment of a test period in the real data is detected as an upward departure from the diagonal. From the QQ plots for 12-, 16-, 24-, and 48-h periods, it is clear that wild-type flies show a dramatic increase in transcript oscillations only at the 24-h period. The range of the null model distributions at any period extends only to include Fourier scores of about 0.4, whereas there are dozens of transcripts with scores above 0.4 in the real data at 24 h. In contrast, the distributions of the real 12- and 16-h oscillatory data are indistinguishable from the null model. Interestingly, there is a modest enrichment in 48-h rhythmicity, a point that will be discussed in detail below.

To further investigate whether the circadian period dominates transcriptional oscillation, we conducted a spectral analysis of data from eight days of time-course experiments each with a 4-h sampling rate (see Materials and Methods). The data were fitted with cosines between 12 and 48 h in period. For each frequency, the number of transcript profiles with at least 30%, 40%, or 50% of their variance accountable by the cosine fit were counted. Figure 2B clearly shows that cosine fits of the data produce the largest sets of well-fitted transcript profiles at periods around 24 h.

These results strongly support the idea that there is extensive control of rhythmic transcription by the circadian clock. They also suggest that circadian expression rhythms are selectively preferred over ultradian or infradian rhythms.

### A Refined Description of the *Drosophila* Circadian Expression Program

To determine the extent of the circadian expression program, we conducted an integrative analysis of all data available from published and unpublished sources. We reasoned that combining microarray data collected from 17 independent days would allow an analysis with greatly increased statistical power. The distribution of 24-h oscillatory statistics in this 17-d integrated dataset (Figure 2C) emphasizes the existence of a substantial circadian expression program.

Marked 24-h periodic trends were also observed in separate analyses for light/dark (LD) time-course data and dark/dark (DD) time-course data. Comparison of the red “LD-only” and green “'DD-only” plots in Figure 2C reveals the enhancement of daily molecular rhythmicity in the presence of an environmental photocycle. This is further quantified in Figure 2D; there are substantially more daily transcript oscillations in the presence of an LD cycle than in constant darkness. There are two possible causes of this observation: (1) it may reflect the improved amplitude and synchrony observed for circadian patterns in LD versus DD conditions, and (2) it might reflect the presence of light-dependent transcript oscillations.

The results of this and other analyses are available in their entirety online (http://flyfits.unil.ch).

### Hundreds of Daily Transcript Oscillations

From the distribution comparison illustrated in Figure 2C, we can make estimates of the total number of 24-h transcript oscillations. If we simply choose the transcripts that exceed the highest quantile value (0.216) from the permuted background model, we would find 148 patterns, but this is likely an underestimate. Alternatively, the enrichment in 24-h periodic patterns relative to the permuted background model is maximal (~750 circadian genes) at a spectral power value cut-off of approximately 0.05, roughly where the plot departs from the diagonal, but this is likely an overestimate. In order to interpret our analysis at the level of individual transcripts, it is important to determine how the reliability of our predictions changes over a range of possible cut-off values. We therefore determined the estimated false discovery rate (FDR) over a range of cut-off value stringencies (Figure 2D). The FDR for a set of putative oscillatory transcripts is defined as the number of transcript profiles that would be selected from randomly permuted data divided by the number of transcript profiles actually selected from the real dataset (see Materials and Methods). This analysis, when applied to the combined LD + DD data, predicted a daily expression component with greater than 95% reliability (FDR < 0.05) for approximately 300 transcripts. Statistical measures representing 24-h rhythmicity in the DD portion of the data (24-h Fourier component and variation with daily time) were then used to verify significant circadian regulation for these transcripts; more than 75% passed one of these requirements at *p* < 0.05, whereas greater than 35% passed both tests. Thus, we reliably identify circadian regulation for more than a hundred transcripts.

### No Circadian Transcripts in Adult Heads of Arrhythmic *tim^01^* Mutant Flies

**Figure 3:**
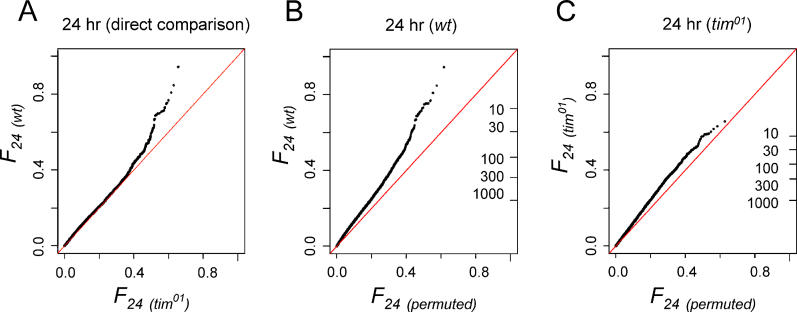
Comparison of 24-h Oscillations between Wild-Type and *tim^01^* Genotypes (A) Direct comparison between value-ordered wild-type and *tim^01^* 24-h Fourier scores. The upward trend represents a clock-dependent enrichment in high-quality circadian oscillations. The same format is used here as for Figure 2A except that the *tim^01^* Fourier scores take the place of the permutation background model. To compare equal numbers of days in both genotypes, four days of data (2× LD/DD) are used on each axis. (B and C) QQ plots comparing the data in panel (A) to a permutation null model (method as in Figure 2A). (B) Comparison of wild-type data to the permutation null model shows that the enrichment in Fig. 2A persists (although somewhat damped) with half the amount of data (2xLD/DD). (C) Comparison of *tim^01^* data (2× LD/DD) to the permutation null model shows that 24-h oscillations, if present, are not obviously enriched in the absence of a working circadian clock.

To measure the extent to which circadian transcription is affected in *tim^01^* animals, we compared time-series data obtained from this mutant to wild-type flies, and both of these to permuted data (Figure 3). A direct comparison between 24-h spectral power *(F_24_)* in wild-type and *tim^01^* (Figure 3A) shows that the enrichment detected in Figure 2A is preserved under the biological null model provided by *tim^01^* data. This demonstrates that LD/DD wild-type data are more rhythmic at the circadian period length than LD/DD *tim^01^* data, without excluding that some rhythms might persist in *tim^01^*. Figure 3C addresses the latter issue, using the same permutation model as in Figure 2A, showing that if at all present, the enrichment is much weaker than that seen using an equal amount of wild-type data (Figure 3B).

**Figure 4:**
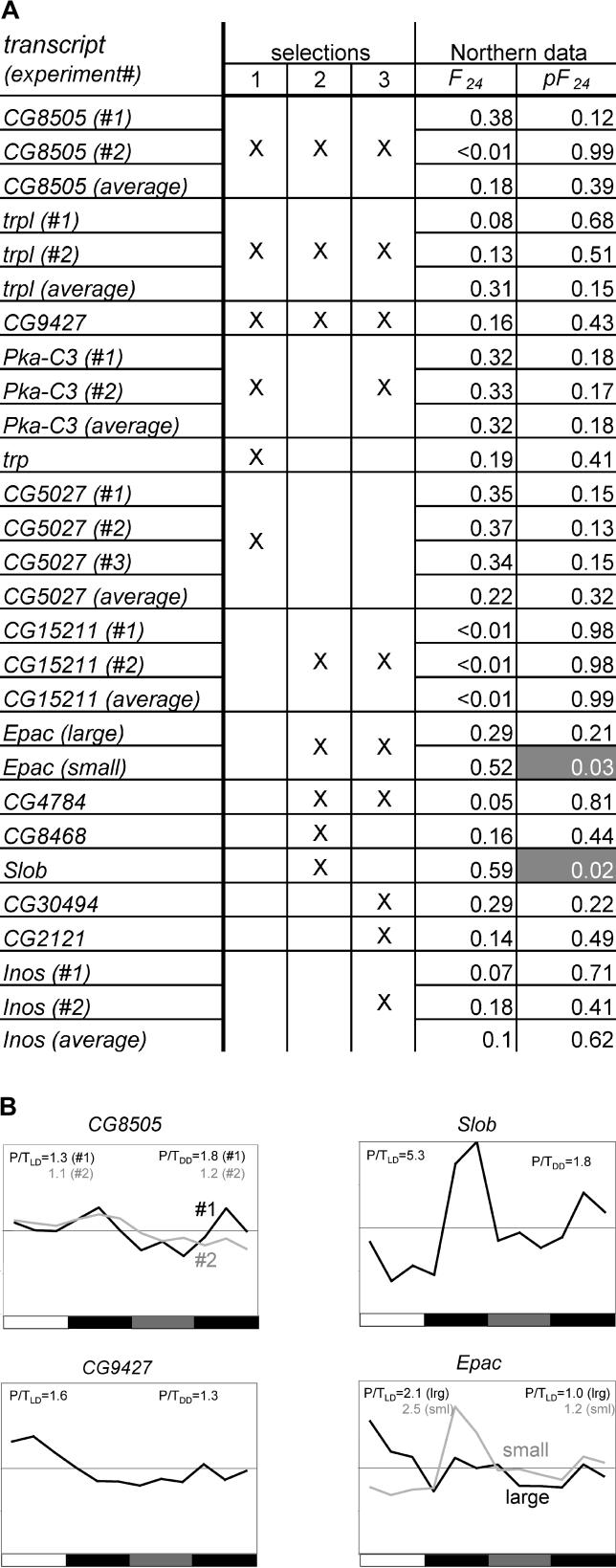
Northern Analyses Confirm the Absence of Circadian Transcript Profiles from *tim^01^* Fly Heads (A) LD/DD time-course Northern blots were performed in *tim^01^* flies for the 14 genes listed in column 1, which were sampled from three selections of candidates with the highest probability of showing circadian oscillations in the *tim^01^* flies. Selection 1 represents a top-down ranking of the 30 best circadian oscillators for the *tim^01^* microarray data as predicted by Fourier analysis. Selection 2 uses the same criterion, but only includes transcripts with a predicted circadian oscillation in wild-type flies. Selection 3 refers to a top-down ranking of the 30 best circadian oscillators for a combined dataset containing an equal number of *tim^01^* and wild-type microarrays. The criteria for Selection 3, but not Selection 2, require that the predicted circadian oscillations for *tim^01^* and wild-type be in the same phase. An X in columns 2–4 indicates which selection(s) each gene is a member of. The 24-h Fourier scores and associated probabilities *(pF_24_)* observed in the Northern profiles are given in columns 5 and 6. Three independent hybridizations were performed for CG5027 and two were performed for CG8505, trpl, Pka-C3, and Inos. In these cases, the statistics for the average profile were also determined. The two distinct transcripts (“small” and “large”) that were observed on Northern blots for Epac were analyzed separately. *pF24* values of ≤ 0.05 are indicated by white script in shaded boxes. Only the *pF24* and *F24* scores for Slob and the smaller transcript of Epac indicate daily oscillations in tim01. All other transcripts showed non-oscillatory expression patterns with high pF24 scores. (B) Further investigation revealed that Slob and Epac show light-regulated rather than circadian responses. Graphed Northern data for five of the fifteen transcripts *(CG8505, Slob,* and *CG9427,* and two transcripts, indicated as “small” and “large” for *Epac)*. Peak to trough expression ratios for the LD and DD parts of the time courses are indicated as P/T. The complete graphed Northern data are available in Figure S1.

Nevertheless, the *tim^01^* data reveal a number of transcripts with *F_24_* scores of approximately 0.7, within the range of values of confirmed oscillatory transcripts in wild-type (Figure 3B and 3C; unpublished data). To systematically test if any reproducible circadian transcript profiles could be detected in the *tim^01^* data, we established three selections of genes with the highest potential for circadian oscillations in the *tim^01^* flies (see Materials and Methods; Figure 4A), and independently tested transcripts by Northern blot analyses (Figure 4A and 4B; Figure S1). High-ranking (within top 30) candidates from each of the three selections were sampled for our independent verification analysis (Figure 4A).

Using Northern expression analysis of *tim^01^* LD/DD time-course experiments, we collected at least one independent expression profile for each of 14 genes. None of these profiles indicated a persistence of circadian regulation (Figure 4A and 4B; Figure S1). The *F_24_* values obtained by Fourier analyses of the Northern data are shown in Figure 4A. With the exception of RNAs expressed by *Slob* (Slowpoke binding protein) and *Epac,* all of the transcripts characterized have *F_24_* values of less than 0.4 (*pF_24_* > 0.1), indicating the absence of significant circadian oscillation. Closer inspection of the results for *Slob* and *Epac* revealed that light-dependent rather than *tim*-independent circadian responses are associated with these genes (as discussed further below). Indeed, during the transition from the LD cycle to the first day of constant darkness, oscillatory amplitude dampens approximately 3-fold for *Slob* and approximately 2-fold for *Epac,* leaving peak-to-trough ratios of 1.8-fold and 1.3-fold, respectively (Figure 4B). Taken together, our results support the hypotheses that: (1) All circadian transcript oscillations observed in the wild-type data depend on a *tim*-dependent circadian clock, and (2) there is no novel circadian rhythmicity in *tim^01^* flies.

### Clock-Dependent and Light-Driven Expression Signatures

**Figure 5:**
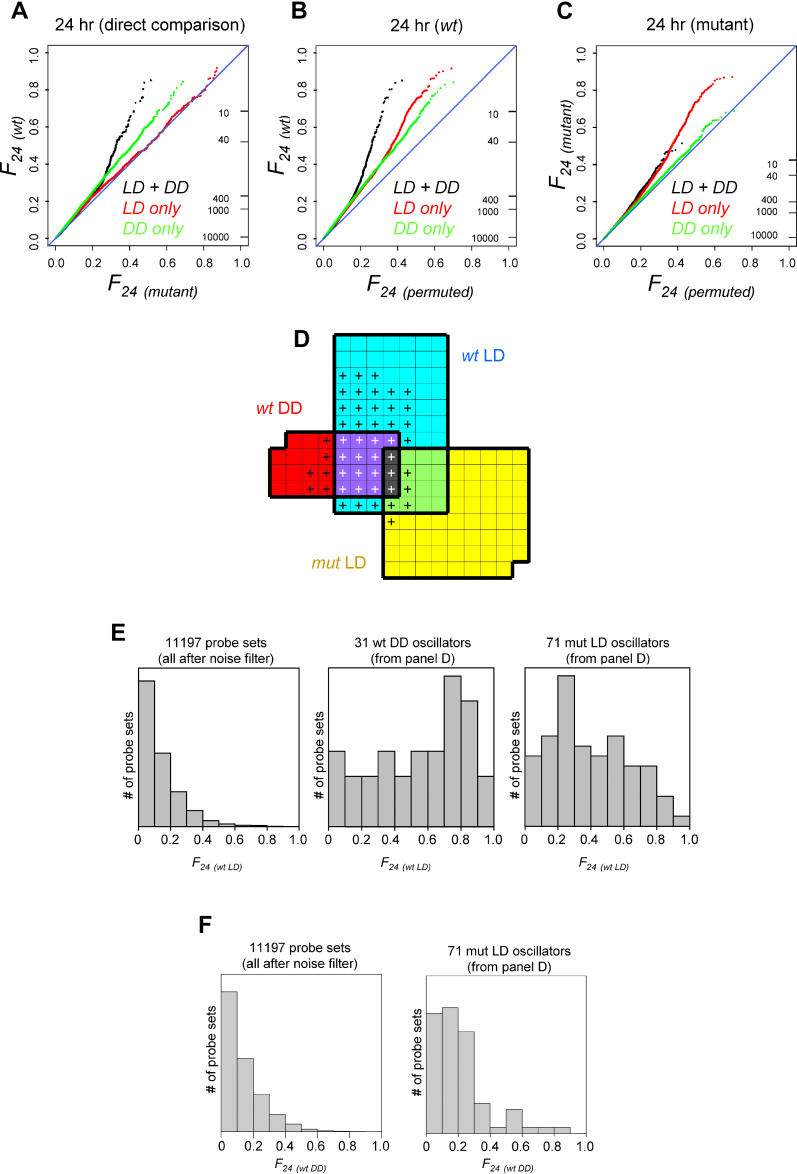
Separate and Combined Effects of Light and the Circadian Clock on Daily Expression Profiles (A) QQ plot (same format as Figures 2 and 3), showing direct wild-type to mutant comparisons of the 24-h spectral power distributions for time-course data obtained in LD conditions (4× 1 d), DD conditions (4× 1 d), or the combined LD and DD datasets. (B and C) The arrhythmic mutant datasets consist of equal amounts *tim^01^* and *per^0^* data. QQ plots that separately compare the wild-type and mutant 24-h spectral power distributions to permuted background models are shown. Clock-independent light-driven effects are responsible for the enrichment in oscillatory profiles in mutant LD (red) versus DD (green) data (C), whereas light-independent circadian effects are represented by the difference between wild-type and mutant DD (green) spectral power distributions (A) and compare (B) with (C). The enhanced difference between the combined LD + DD datasets (black) for wild-type and mutant, visualized by the deviation from the diagonal in (A), indicates the alignment of LD and DD rhythmic profiles that occurs as a result of clock-dependent regulation. (D) Circadian and light-driven regulation is indicated by overlap among the top-ranking oscillators in wild-type LD, wild-type DD, and mutant LD. The diagrammed selections of 77 wild-type LD profiles, 31 wild-type DD profiles, and 71 mutant LD profiles were obtained by demanding a high 24-h spectral power (*p* ≤ 4.5 × 10^−4^ [approximately *F_24_* > 0.6]) and applying two noise filters (more than half of the values above the 20th percentile and absolute range > 0.5). No mutant DD profiles were found that passed these selection criteria. Circadian regulation is indicated by the significant overlap between the wild-type LD and wild-type DD selections (Fisher's exact test *p* < 10^−26^), whereas light-dependent regulation is represented by the significant overlap between the wild-type and mutant LD selections (Fisher's exact test *p* < 10^−19^). The plus signs (+) correspond to transcript profiles that rank among the best oscillators in the wild-type LD + DD dataset (*p* ≤ 10^−4^; more than half of the values above the 20th percentile; absolute range > 0.5). (E) Wild-type LD spectral power scores are dramatically increased among wild-type DD oscillators and mutant LD oscillators. Histograms of the wild-type LD 24-h spectral power are shown from left to right: for all transcripts exceeding a noise filter (11,197 with more than half of the values above the 20th percentile), and for the selections of 31 wild-type DD oscillators and 71 mutant LD oscillators from panel (D). Mann-Whitney rank sum tests confirm that these differences are highly significant (*p* < 10^−13^ for 31 wild-type DD oscillators versus rest; *p* < 10^−24^ for 71 mutant LD oscillators versus rest). (F) Wild-type DD spectral power scores are elevated among mutant LD oscillators. Histograms of the wild-type DD 24-h spectral power are shown on the left for all transcripts exceeding a noise filter (11,197 with more than half of the values above the 20th percentile) and on the right for the selection of 71 mutant LD oscillators from panel (D). A Mann-Whitney rank-sum test confirms the significance of this difference (*p* < 10^−4^).

As mentioned above, we detected modest 48-h rhythmicity in wild-type LD/DD time-course data (see also Figure S2). This observation, along with the apparent rhythmicity of a few transcripts in LD/DD *tim^01^* data, led us to believe that the inclusion of LD data in our analyses might introduce light-driven oscillations. The presence of oscillations driven by cycling light was also suggested by the increased rhythmicity in LD data when compared with DD data (Figure 2C and 2D). To verify the existence of light-driven rhythms, we compared genome-wide 24-h periodicity in separate LD-only and DD-only datasets for wild-type and arrhythmic *per^0^ (period^0^)* and *tim^01^* mutant flies (Figure 5). Comparison of separate wild-type LD and DD spectral power distributions exposes the stimulation of daily transcript oscillations by the environmental LD cycle (Figure 5B). When the analogous comparison for arrhythmic mutant flies is performed, the DD-only plot confirms the absence of a circadian clock, but the LD-only plot demonstrates the presence of a broad program of light-driven oscillations that occur independently of a functional clock (Figure 5C). This program of light-driven gene regulation is so large that in an LD context, the daily periodic trends observed for wild-type and arrhythmic mutant flies are approximately equal in strength (Figure 5A, LD-only red plot). This is solely an effect of the Zeitgeber light conditions because oscillations that persist in DD are specific to wild-type flies (Figure 5A–C and 5D; see also Figure 3C and 4, above). The amplified qualitative difference in 24-h periodicity between the integrated LD + DD datasets for wild-type and mutant flies (deviation from the diagonal in Figure 5A; compare LD + DD with DD only) indicates that synergism between clock- and light-dependent regulation exists in wild-type flies but not in arrhythmic mutant flies.

### Overlap of the Circadian and Light-Regulated Transcriptional Programs

We aimed to examine the relatedness of transcript oscillations found in Zeitgeber LD conditions with those found in DD circadian conditions. From each of wild-type LD, wild-type DD, mutant LD, and mutant DD conditions, the top-ranking oscillators were selected and the overlap between selections was examined. Figure 5D illustrates the large degree of overlap between wild-type LD and wild-type DD oscillators, a highly significant (Fisher's exact test *p* < 10^−26^) and unsurprising result, considering the extensive program of circadian expression that is detected in combined wild-type LD + DD data (Figures 2A, 2C, 3A, and 5A). In addition, clock-independent light responses are represented in Figure 5D by the 16 transcript overlap (*p* < 10^−19^) between the wild-type LD and mutant LD oscillator selections. These circadian and light-driven trends are also evident from the comparative histograms of Figure 5E, which illustrate the enhanced wild-type LD periodicity associated with wild-type DD and mutant LD oscillations, respectively. Interestingly, there is significant overlap between light- and clock-regulated genes. The set of mutant LD oscillators from Figure 5D shows an increase in circadian expression components observed in a wild-type DD context (Figure 5F, Mann-Whitney rank sum test *p* < 10^−4^). Indeed, there is a class of genes that show both strong light-driven and strong circadian responses. This class is represented in Figure 5D by the three genes that rank among the top oscillators for wild-type LD, wild-type DD, and mutant LD conditions (three-way overlap), and by the five additional genes that show strong 24-h periodicity in both mutant LD data and combined wild-type LD + DD data (other genes in the mutant LD selection marked with a plus [+] symbol).

### Identification of Light-Induced and Light-Repressed Transcripts

**Figure 6:**
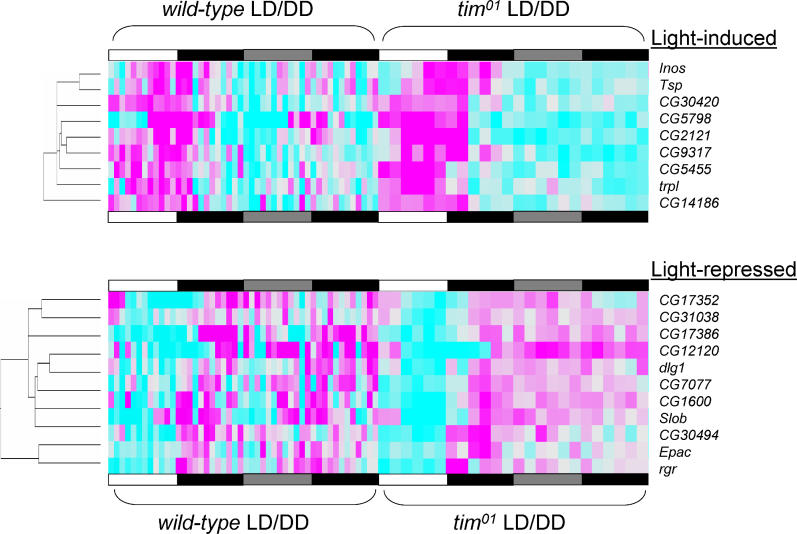
Transcripts with a Sustained Photoresponse A selection of 20 light-driven transcripts emerges from statistical analysis of wild-type and *tim^01^* microarray expression data (see Materials and Methods). Pairwise hierarchical clustering of the expression profiles for this selection indicates a grouping of nine light-induced transcripts and a grouping of 11 light-repressed transcripts. Columns correspond to experimental time points, rows correspond to genes. Colors in the order cyan to light gray to magenta represent normalized RMA values of increasing strength, with light gray corresponding to the time-course average. The blocked horizontal bars below the cluster diagrams illustrate the environmental light/dark schedule used in the time courses, with white indicating light, black indicating darkness, and gray indicating subjective light under free-running conditions of constant darkness. The first 48 columns in the cluster diagrams represent the RMA expression values normalized per experiment and ordered by experimental time for four wild-type time courses, whereas the last 24 columns represent the two *tim^01^* time-course experiments in the same format. The trees on the left of the cluster diagrams show the pairwise similarity relationships between the clustered transcript profiles. Note the almost purely light-driven patterns in *tim^01^* flies and the more complex patterns observed in a wild-type context where there is often also a circadian expression component present.

We selected a set of the most reliably light-dependent transcript oscillations so that they could be studied in greater detail, including verification by Northern blot. First, we identified putative light-regulated genes based on their microarray profiles. Transcript profiles were included in our selection if they (1) demonstrated high 24-h Fourier spectral power and 24-h autocorrelation in combined wild-type LD + *tim^01^* LD data, (2) showed differential expression between the light phase of an LD cycle and the “subjective light phase” of the first day of free-run in constant darkness, and (3) passed our noise filters (see Materials and Methods for details). This strategy was used to identify the 20 putative light-driven transcript profiles described in Figure 6. Hierarchical cluster analysis divides this group into a subset of nine light-induced transcripts and a subset of 11 light-repressed transcripts. Closer inspection of the cluster diagrams in Figure 6 reveals that the *tim^01^* time-course expression patterns present themselves as purely light-driven profiles, whereas the wild-type time-course expression patterns for many genes (e.g., *CG5798, CG2121, Slob,* and *CG17386*) appear to have both light-dependent and circadian components.

### Northern Blot Analysis Verifies Widespread Light-Driven Gene Expression

**Figure 7:**
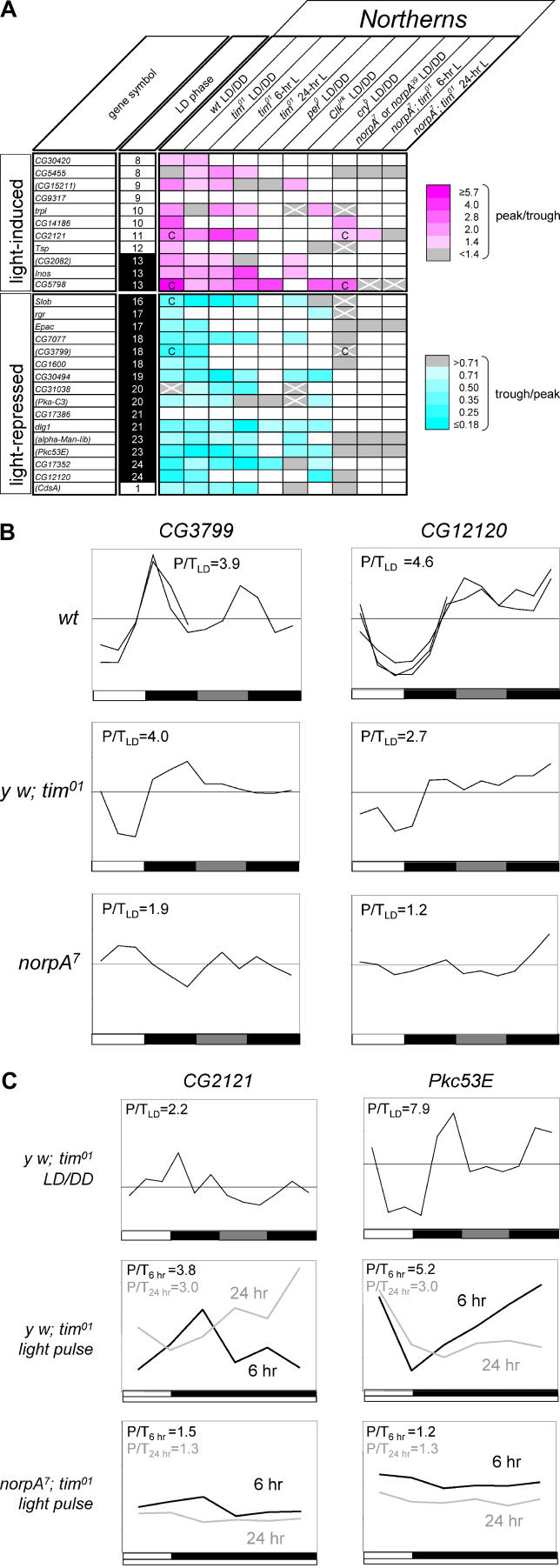
Expression Analysis of Light-Regulated Transcripts (A) Northern analyses confirm light-driven regulation for 25 transcripts. This regulation persists for most transcripts in the context of circadian clock mutants *(tim^01^, per^0^,* and *Clk^jrk^)* or the circadian photoreception mutant *cry^b^,* but it is disrupted by mutation of the *norpA* gene. Light-regulated transcripts were identified based on statistical analysis of microarray data. For completeness, the group of 20 from Figure 6 is supplemented here with seven additional light-regulated transcripts *(CG15211, CG2082, CG3799, Pka-C3, alpha-Man-IIb, Pkc53E,* and *CdsA)* that emerged from our selection protocol when an alternative microarray signal algorithm was used (MAS 4.0 instead of RMA). Rows corresponding to individual transcripts (column 1) are ordered based on the peak expression phase (in 1-h intervals of ZT, relative to ZT0 = lights-on), estimated by 24-h spectral analysis of wild-type LD microarray profiles (column 2). As a result, the light-induced transcripts (rows 1–11) are separated from the light-repressed transcripts (rows 12–27). Northern analysis results are summarized for 2-d LD/DD time-course experiments with wild-type, *tim^01^, per^0^, Clk^jrk^, cry^b^,* or *norpA* mutant flies (columns 3, 4, and 7–10) and 1-d time-courses describing 6-h or 24-h light-pulse treatments of dark-raised *tim^01^* or *norpA^7^; tim^01^* flies (columns 5, 6, 11, and 12). The peak/trough ratios for light-induced genes and trough/peak ratios for light-repressed genes that were observed during the LD portion of LD/DD time courses (columns 3–4 and 7–10) or the 1-d light pulse experiments (columns 5–6 and 11–12) are color-coded as indicated, with light-induction and repression represented by increasingly intense shades of magenta and cyan, respectively. Gray rectangles represent patterns with peak/trough ratios below 1.4 or trough/peak ratios above 0.71, and gray rectangles with white crosses correspond to other patterns inconsistent with light-driven regulation. Combined analysis of Northern and microarray DD time-course data indicated exceptionally strong circadian regulation (*F_24_ p-value* < 10^−3^; indicated as “C”) for four of the light-regulated transcripts (*CG2121, CG5798, CG3799,* and *Slob;* column 3), and this regulation persisted in a *norpA* mutant context for three of these (*CG2121, CG5798,* and *CG3799;* indicated as “C” in column 10). (B and C) Examples of the Northern data summarized in panel (A). Graphed Northern analyses for four light-regulated transcripts. In panel (B), wild-type, *y w; tim^01^,* and *norpA^7^* graphed LD/DD time-course profiles are shown for two light-repressed transcripts, *CG3799* and *CG12120,* whereas panel (C) represents light responses observed in a *y w; tim^01^* LD/DD time course and in light-pulse treated *y w; tim^01^* or *norpA^7^; tim^01^* flies for one light-induced transcript *(CG2121)* and one light-repressed transcript *(Pkc53E)*. After background subtraction, Northern signals for LD/DD time courses were normalized to a loading control and graphed relative to the normalized time-course average (indicated as a horizontal). One-day LD time courses (indicated by the shorter lines in the wild-type panels) are centered on the average expression ratio observed during the LD part of matching LD-DD experiments. The combined 6-h and 24-h light pulse profiles for *y w; tim^01^* and *norpA^7^; tim^01^* were normalized to the average of the ZT2 and the ZT6 time points that were taken during both light pulse treatments. Peak to trough expression ratios for the LD part of time courses or the 1-d light-pulse experiments are indicated as P/T. Each line represents the results from a separate blot. The complete graphed Northern data for light-driven transcripts is available in Figure S3.

The predicted light-driven transcript profiles of Figure 6 were independently verified using Northern analysis of wild-type and mutant data, and the results are summarized in Figure 7A. Data for an additional seven confirmed light-regulated transcripts *(CG15211, CG2082, CG3799, Pka-C3, alpha-Man-IIb, Pkc53E,* and *CdsA)* that were not part of the selection described in Figure 6 but emerged from a similar selection strategy using an alternative microarray signal algorithm (MAS 4.0 instead of robust multi-array average [RMA]) are also included in Figure 7A. The light-driven transcripts in Figure 7A are ordered according to their estimated peak-expression phase during a photocycle in wild-type flies (Figure 7A, column 2), which also has the effect of separating light-induced and light-repressed transcripts. Light-dependent responses were verified using Northern analysis in an LD/DD time-course format for both wild-type and *tim^01^* flies. We provide confirmatory evidence of light-dependent regulation in wild-type flies for 23 transcripts (Figure 7A; column 3; Figure 7B; Figure S3). For each of these transcripts, we observe wild-type expression profiles that show the predicted pattern of up- or down-regulation in an environmental photocycle. For a subset of four of these transcripts, the wild-type Northern data indicate a particularly strong circadian expression rhythm (*CG5798, CG2121, Slob,* and *CG3799;* Figure 7A, column 3). Light-regulated expression patterns are also observed in the context of the *tim^01^* arrhythmic mutant (Figure 7A, columns 4–6; Figure 7B and 7C). With the exception of *trpl* (trp-like), all 22 transcripts included in this analysis respond to the photocycle in the first half of the *tim^01^* LD/DD time-course experiment (Figure 7A, column 4; Figure 7B and 7C).

### Separate Clock and Photocycle Components in Transcript Profiles

**Figure 8:**
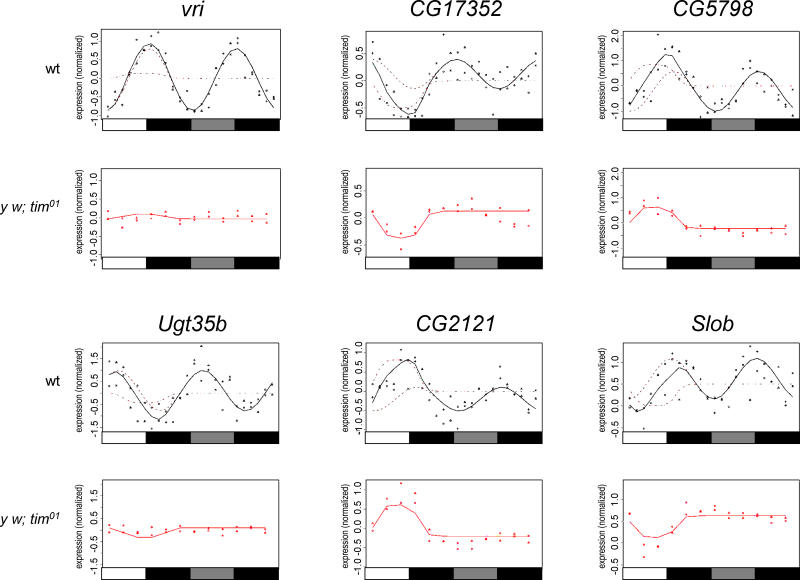
Superposition of Photocycle and Circadian Expression Patterns Revealed by a Parametric Model Normalized RMA expression ratios for the wild-type LD/DD time-course data are fitted to a model consisting of a cosine function representing circadian expression and a pulse function representing light-dependent regulation. During the LD conditions of the first 24 h, both functions contribute to the fit (as indicated by the dotted lines), but during the DD conditions of the second day, the model reverts to the cosine function. *tim^01^* LD/DD expression ratios are fitted to a pulse-only model. Examples are shown for two standard circadian genes *(vri* and *Ugt35b)* and four light-regulated circadian genes *(rgr, CG2121, CG5798,* and *Slob)*.

Many of the light-driven transcripts documented in this study show profiles with both light-dependent and circadian tendencies in wild-type flies (Figure 6; Figure 7A, column 3; Figure 7B). In arrhythmic *tim^01^, per^0^,* or *Clk^jrk^* (Clock^jrk^) mutant flies, however, LD/DD time-course profiles for these transcripts are reduced to approximate on/off patterns for light-induced and off/on for light-repressed genes (Figure 6; Figure 7B). Because circadian expression profiles can be successfully represented as 24-h periodic sine waves, we built a model (see Materials and Methods) in which a clock-driven cosine function and a light-dependent rectangular function independently contribute to the expression profile. All genes can be viewed at http://flyfits.unil.ch. Several examples are shown in Figure 8. Although it is clear that this simple model can account only for a subset of possible time traces, it accurately captures three types of regulation: (1) light-driven expression, (2) circadian expression, and (3) combined light-driven/circadian expression.

### Light-Driven Regulation in *per^0^* and *Clk^jrk^* Mutants

We conducted LD/DD time-course Northern analyses for two additional arrhythmic mutants, *per^0^* and *Clk^jrk^*. The *per^0^* mutant, like *tim^01^,* arrests the clock in a state where the core clock transcription complex CLK/CYC (clock/cycle) is constitutively active, whereas the *Clk^jrk^* mutant has the opposite effect (for review, see Hardin [4]; Hall [2]). We tested a total of 15 light-regulated transcripts in at least one of these mutant backgrounds, and 11 of these showed partial or complete persistence of the light-dependent regulation. Persistence of light responses was detected in three out of five cases for the *per^0^* mutant and nine out of 14 cases for the *Clk^jrk^* mutant (Figure 7A, columns 7 and 8). Although these results confirm that the light-dependent responses that we identified generally do not require a functional circadian clock, they also indicate that at least some of them are affected by the state of specific clock genes.

### Tracking of Environmental Photoperiod by Light-Driven Expression Responses

Our microarray and Northern LD/DD time-course analyses for light-regulated transcripts suggested that they show slow, but sustained expression responses to light. We reasoned that these were primary responses that did not require multiple preceding days of photocycle. To test this hypothesis directly we analyzed light-dependent expression responses in dark-raised *tim^01^* flies that were given a single light pulse of either 6 or 24 h in length. All surveyed transcripts (17) responded to this type of light treatment (Figure 7A, columns 5 and 6; Figure 7B and 7C; Figure S3). Moreover, in most cases (12 of the 17), the duration of the expression response obviously matched the length of the experimental photoperiod (6-h pulse, 12:12 LD cycle, or 24-h pulse; Figure 7C; Figure S3). Thus, although we initially identified this light-driven effect as an oscillation, it would only be so under photocycling LD conditions. Rather, these genes are truly light-driven, responding to light exposure directly.

### The Roles of Known Circadian Photoreceptors in Light-Driven Responses

The two major pathways for entrainment of the circadian clock to light involve photoreception by CRY (cryptochrome) in clock-bearing cells, and phototransduction in the light-sensing organs [30,31]. Time-course Northern blots on LD/DD *cry^b^* mutant fly heads showed that light-dependent regulation persisted in *cry^b^* flies for nine of eleven transcripts tested (Figure 7A, column 9). The amplitudes and durations of these sustained responses were similar to those observed in *tim^01^* flies. Although *Tsp* showed a mild decrease in photocycle amplitude, *Slob* was the only gene for which light-dependent regulation appeared to be completely dependent on CRY function (Figure S3). In light of the minor role of CRY, we turned our attention to the role of the light-sensing organs. Since *norpA,* which encodes the phospholipase C component of the visual transduction pathway, had been shown to play a key role in mediating CRY-independent photo-entrainment [30], we conducted two experiments addressing its potential involvement in mediating light-driven transcript responses. In the first experiment we studied the expression profile of 16 light-regulated transcripts in a synchronous population of *norpA^7^* or *norpA^39^* flies during a single LD photocycle and a subsequent day in constant darkness (see Materials and Methods). Thirteen of these transcripts clearly require NORPA for their light-driven behavior, whereas the results for two of the remaining three transcripts were inconclusive due to the presence of synchronous circadian regulation *(CG2121* and *CG5798)*. In a second experiment, we measured mRNA expression profiles in dark-raised *norpA^7^; tim^01^* flies that received a single light pulse of either 6 or 24 h in length. The light pulse–induced responses observed in *tim^01^* flies were clearly decreased in the *norpA^7^; tim^01^* double mutants. In five out of six cases, the responses were not detectable at all (Figure 7A, see columns 5 and 6, and 11 and 12). Over all, there were only two instances in which we found possible residual light responses in a *norpA* mutant context. The *norpA^39^* LD/DD time course for *CG14186* showed a somewhat enhanced fluctuation in LD when compared to DD and there was a marginal 6-h light pulse response in *norpA^7^; tim^01^* flies for *CG2121*. However, even in these two cases, the light responses were clearly reduced in the absence of NORPA (Figure 7A; Figure S3). Taken together, our results suggest that photocycle-dependent transcript oscillations depend on NORPA. To address the question whether NORPA carried out this function in the adult compound eyes, we compared the expression levels of light-regulated genes in the heads of *eya^2^* mutant flies, which lack the compound eyes, to those in wild-type flies using microarray analysis. For all light-driven genes described in Figures 6 and 7, the average expression signal observed in *eya^2^* mutant heads was lower than that found for wild-type controls, and this trend was highly statistically significant (see Figure S4). Moreover, the fact that the expression signal for a number of confirmed light-driven genes in *eya^2^* did not exceed background noise (unpublished data) indicates that our observations of light-dependent regulation of these genes in wild-type heads indeed reflects regulation in the compound eyes. Thus, at least some of the light-dependent regulation that we identified apparently occurs downstream of the phototransduction pathway in the compound eyes.

## Discussion

### A New Strategy for Identifying Oscillatory Trends in Microarray Data

In recent years, five different sets of circadian transcripts have been proposed for the *Drosophila* head in as many publications [15–19]. Unfortunately, the overlap between these transcript sets is very poor (seven transcripts), and it falsely excludes numerous confirmed circadian transcript oscillations. These recent genome-wide surveys for rhythmic transcription have defined groups of circadian transcripts based on empirical ranking and filtering approaches, often using necessarily arbitrary cut-offs. To complement these studies we developed a method for examining periodic expression at the systems level, allowing us to pursue a number of new investigations. This new strategy enabled us to describe the programs of circadian and light-driven transcription in the adult fly head. Because our method emphasizes uniformity in period length and peak phase while tolerating inter-experimental variability in amplitude, it is particularly successful at measuring oscillatory trends across different independent experiments. Integrative analysis of all available microarray time-series data allowed detection and ranking of oscillatory transcript profiles with improved resolution and revealed a circadian expression program that is much more substantial than the apparent consensus (or lack thereof) between different published studies indicates. Some of the best described and strongest circadian oscillations *(per, Clk, Pdp1, cry,* and *to)* were missed in one or more of the previously published studies, but all of these rank high in our integrative analysis. Although there are relatively few genes (~50, unpublished data) that show the same level of circadian regulation as the oscillating components in the core clock circuits *(per, tim, Clk, cry, vri,* and *Pdp1)*, our results provide evidence for a substantially broader circadian expression program downstream of the core oscillator. This suggests that the circadian clock is responsible for both the purely circadian expression patterns of a limited set of genes and the partial circadian regulation of a much greater group.

### One Transcriptional Clock in *Drosophila*

Whereas many of the genes composing the *Drosophila* clock are expressed with a circadian rhythm in wild-type flies, all known clock gene oscillations cease if just one of them is lost by mutation (reviewed in [2,3,8,9]). We reasoned that all of the circadian oscillations in gene expression that we identified should stop in *tim^01^* mutants if these were truly devoid of a circadian clock. Alternatively, rhythmicity could theoretically persist in a subset of the genes if their expression depended on a parallel, novel circadian clock. Our distribution analyses allowed us to address these two alternative possibilities. No alternative systems of oscillatory expression are detectable for the 12–48-h range of period lengths. In the absence of *tim*-dependent clock circuits, no circadian patterns of gene expression were detected. This latter result, from our microarray and Northern analyses, is in agreement with earlier observations, with limited sampling of individual circadian transcripts [32–38]. Moreover, the absence of detectable molecular circadian rhythms fits well with the abolition of circadian eclosion and locomotor rhythms in *tim^01^* flies [28]. Thus, *Drosophila* appears to possess only one, *tim*-dependent, circadian clock. This observation contrasts with results from cyanobacteria, protists, fungi, and plants that suggest the presence of multiple oscillators, sometimes even in the same cell; reviewed in [39,40]. Although there is no compelling evidence supporting the existence of alternative circadian clocks in *Drosophila* that are not entrainable to light or independent from transcriptional rhythms, we admit that formally our study does not disprove these possibilities. Our results complement and extend previous microarray and differential display analyses using different arrhythmic mutants *(per^0^* or *Clk^jrk^)* in which few [15,17,19] or apparently no [18] daily transcript oscillations persisted in the mutant context.

### A New System of Light-Regulated Gene Expression in *Drosophila*

Comparative analysis of data collected from wild-type and arrhythmic mutant flies in the presence or absence of an environmental photocycle allowed us to identify a program of light-driven regulation. The *tim^01^* mutant flies used for these experiments do not just have a defective circadian clock, but because TIM degradation is a major mechanism of clock re-setting, they have also lost the main photic input pathway that entrains the clock circuits to light. In a wild-type context, light can directly entrain clock-bearing tissues in a cell-autonomous manner by activating the circadian photoreceptor CRY, or it can entrain the pacemaker neurons in the brain via phototransduction in the visual organs [30]. TIM is the target for CRY's effect on the clock circuits [41–43], and it may also play a role in mediating entrainment via the visual organs [44]. In spite of their defective clock circuits and circadian entrainment pathways, *tim^01^* mutant flies retain an extensive set of daily transcript oscillations in the presence of an environmental photocycle. By comparing the light-driven expression signature that we found for *tim^01^* with the microarray analysis for *per^0^* LD by Lin and colleagues [17] and with our confirmatory northern analyses (Figure 7A; Figure S3), we have established that many light-driven transcripts show the same expression profiles in *per^0^* and *tim^01^* arrhythmic mutants. Moreover, the light-driven expression response found in a combined *per^0^* and *tim^01^* LD microarray dataset is comparable in size to the clock-dependent circadian expression program (Figure 5C).

Light-regulated genes fall into two classes, a clock-independent class, and a group of genes that are also clock-controlled. That there are clock-independent patterns of light-regulated gene expression suggests that coordinate clock- and light-control can be disadvantageous in some circumstances. For example, although the clock carries phase information about the photocycle, it may not be able to carry information about day length and sunlight intensity, and some photoprotective functions might be better linked to acute light activation so that they are delivered only when needed. Such a case might be made for ultraviolet-induced melanogenesis in human skin. In contrast, we suspect that many genes controlled by light and the clock contribute to processes that require both daily anticipation of changes in light and light responsiveness.

### Light-Dependent Control of Expression in Other Organisms

Previous studies of light-regulated gene expression have been carried out for cyanobacteria, protists, plants, fungi, and animals. Light-dependent signaling is particularly important in photosynthetic organisms. In the obligate photoautotrophic cyanobacteria *Synechococcus elongatus,* light-dependent transcription affects practically the entire genome and is coupled to the circadian clock (for a review, see [45]). The chloroplast genome in the green alga, *Chlamyomonas reinhardtii,* may be regulated in a similar fashion [46], although in this system much of the light-dependent regulation of expression takes place at the level of translation [47]. In plants, all stages of development are affected by light-induced gene expression (for a review, see [48]). At least three different photoreceptor systems are used by plants. Phytochromes mediate responses to red/far-red light, cryptochromes and phototropins are responsible for blue/UVA-A light signaling, and UV-B light is sensed by a photoreceptor that has yet to be identified. A model for the program of light-dependent transcription in plants is emerging from studies dissecting photic signaling pathways (e.g., [49–51]) and from genome-wide expression analyses (as reviewed in [52]). Light-dependent expression in fungi and animals has not yet been explored as thoroughly. Individual examples of light-regulated gene expression have been previously reported for *Neurospora* [53,54], *Xenopus* [55], zebrafish [56–58], and mammals [59–63]. One apparent difference between our genome-wide analysis in *Drosophila* and observations of individual light-responsive transcripts in other organisms is that we have not uncovered evidence for light-dependent control of genes encoding known components of the circadian clock. A possible explanation for this discrepancy is that by conducting our analyses with *tim^01^* mutant flies, we have chosen to identify light-regulated transcripts that do not require a working clock or the TIM-dependent photic input pathway. In a wild-type context, phase-resetting of the clock with a light pulse during the early night leads to degradation of cytoplasmic TIM and a relative increase in the levels of *per* and *tim* mRNA, whereas a light pulse during the late night causes degradation of nuclear TIM and has the opposite effect on *per* and *tim* expression [64–66]. Other CLK/CYC-induced transcripts are likely to show behavior similar to that of *per* and *tim* in response to light pulses. Given the light-entrainable properties of the circadian clock all circadian transcripts will ultimately respond to photic input, but depending on their distance from TIM in the regulatory circuits of the clock they may adjust earlier or later in response to a phase-resetting light pulse. In *Neurospora,* transcription of the clock gene *frq* is induced by light via the photo-receptive white collar-1/white collar-2 transcription complexes, and this response is intimately connected to photo-entrainment of the molecular clockworks [54]. In the mouse, *mPer1* may have a similar role in resetting the clock, because it is activated in response to light in the suprachiasmatic nucleus (SCN). However, this is gene activity stimulated by local release of glutamate, and is therefore a secondary response to light that is absorbed by the retina [59,61–63]. Mice homozygous for the phosphorylation site mutant CREB^S142A^ mutation exhibit behavioral defects in photic resetting that are combined with a molecular defect in the light-responses of *c-Fos* and *mPer1,* but not the *mPer2* transcript [67]. The *Xenopus* homolog of *mPer2, xPer2,* does appear to be activated by light in the retina [68], and may therefore encompass a mechanism for light regulation of clock gene expression that is distinct from that found in the mammalian SCN. In transparent zebrafish larvae or zebrafish cell lines, the clock gene homologs *zfPer2* and *zfClock* are induced by light [57], whereas *zfPer4* is light-repressed [58]. We were able to distinguish light-dependent from circadian responses by conducting experiments with arrhythmic mutant *Drosophila*. A similar strategy was followed in the studies describing the light-induction of Neurospora frq and *eas* [53,54]. Maximal induction of mouse *mPer1* and *mPer2* mRNA levels in the SCN occurs relatively soon after exposure to a light pulse (1 and 2 h, respectively), but this response depends on circadian phase [61] and is diminished in the *Clock* mutant [69], suggesting that other clock components are needed. It has not yet been investigated whether a functional clock is required for the light responses observed in *Xenopus* and zebrafish.

### Light Regulation of Gene Expression in the Eye

A survey of published expression studies for our selection of light-regulated genes indicates that many of them are prominently expressed in the adult compound eyes (*trpl* [70]; *CdsA* [71]; *Pkc53E* [72]; *dlg1* [73]; *Slob* [74]; *CG17352, CG5798, CG7077, CdsA, dlg1, Slob,* and *trpl* [75]). Indeed, comparative transcript profiling studies of wild-type and *eya^2^* mutant flies predict expression in the adult compound eyes for 22 of the 27 light-dependent transcripts (Figure S4).

Two of the confirmed light-regulated transcripts *(trpl* and *CdsA)* encode known regulators of phototransduction. We and others observed daily oscillations in the transcript levels for *trpl,* which encodes a light-activated calcium channel [15,16,19,70]. Although some effects on light-activated conductance have been observed in a *trpl* null mutant, the major light-dependent cation channel in *Drosophila* appears to be encoded by its homolog *trp* (transient receptor potential) [76]. Instead, the TRPL protein may have a specific function in phototransduction during extended illuminations and for adaptation of the light response to dim background light [77,78]. The effect of TRPL on long-term adaptation is thought to be mediated via light-dependent subcellular translocation of TRPL protein, resulting in a preferred localization at the photoreceptor membranes in the dark and in the cell-bodies in the light [77]. Experiments in the blowfly Calliphora vicina indicate that this translocation does not require regulation at the transcript level [77], but it is possible that the daily evening peaks of the *trpl* transcript in *Drosophila* facilitate efficient accumulation of TRPL protein at the rhabdomeres around dusk. Daily fluctuations are also exhibited by the transcript for *CdsA* (CDP diglyceride synthetase). The CDSA protein is localized to photoreceptor neurons and catalyzes the synthesis of CDP-diacyl glycerol from phosphatidic acid and CTP [71]. This enzymatic function helps generate the signaling compound phosphatidyl inositol 4,5-bisphosphate, which is consumed during phototransduction by the phospholipase C NORPA. Studies of *CdsA* loss-of-function and gain-of-function mutants indicate that by controlling availability of phosphatidyl inositol 4,5-bisphosphate, CDSA expression levels affect the gain of the phototransduction response [71,79]. Periodic variation of *CdsA* expression under influence of the environmental photocycle could, therefore, be hypothesized to promote daily variations in visual sensitivity.

### Light Effects on Synaptic Gene Expression

Two other light-driven transcripts, *dlg1* and *Slob,* are associated with the regulation of synaptic transmission. The *dlg1* (discs large 1) gene has roles in control of cell growth and differentiation as well as synaptic function [80,81]. DLG1 spatial expression pattern includes synaptic sites in the adult brain and the outer membrane of photoreceptors, where it localizes *Sh* (Shaker) potassium channels [73,82,83].

*Slob* is negatively regulated by light in a clock-independent manner in addition to being one of the most robustly oscillating circadian transcripts in the adult head [15–19]. The clock-dependent and light-dependent fluctuations that we uncover for the *Slob* transcript are reflected in the SLOB protein levels observed in photoreceptor cells and whole heads [74]. A number of findings point to a possible role for SLOB in mediating overt behavioral rhythms. SLOB protein is thought to bind the SLO and EAG potassium channels, and can directly enhance SLO activity, as well as mediate the inhibitory effect of 14–3–3ζ on SLO [84,85]. *slo* mutants have altered potassium channel currents and reported defects in flight, male courtship, and circadian locomotor behavior, whereas mutations of *eag* display hyperactivity, and affect potassium currents and courtship behavior [15,86–92].

### Light-Driven Gene Regulation Requires Phospholipase C

As mentioned above, circadian rhythms in adult *Drosophila* can be entrained to a LD cycle via either opsin-mediated photoreception in the light-sensing organs (compound eyes, ocelli, and eyelets) or cell-autonomous activation of the circadian blue-light photoreceptor CRY [30,31]. Interestingly, the contribution of visual photo-transduction to circadian photo-entrainment is apparently restricted to a few pacemaker neurons in the brain, a situation reminiscent of photo-entrainment of the clock circuits in the mammalian brain via the retina and the retino-hypothalamic tract. In contrast, *Drosophila* CRY contributes to photo-entrainment in many more clock-bearing tissues, including the visual organs [30]. CRY mediates the light-dependent degradation of TIM [42], which in turn affects CLK/CYC transcriptional activity in a manner that depends on the phase of the circadian cycle [64,65,93,94].

The light-driven transcript responses identified in this study resemble circadian responses in amplitude and duration in the context of a photocycle, and are found for a number of genes with a verified circadian expression profile. We, therefore, asked whether these light-driven transcript responses depend on the same light sensors as the circadian system. We found that for the most part light-driven regulation does not require CRY. Given TIM's status as a target for CRY-mediated light responses, it is perhaps not surprising that light-driven expression responses that do not require TIM function also persist in the absence of CRY. There is one interesting exception to this rule: The light-mediated repression of the *Slob* transcript apparently requires CRY, but not TIM. If this observation indeed represents a previously unappreciated function for CRY, it may share this role with the phospholipase C enzyme NORPA, as *norpA* mutants similarly affect the *Slob* transcript.

In contrast with CRY, we found that NORPA phototransduction mediates many if not all of the other clock-independent light responses identified in this study. Based on the overlapping expression of both NORPA and its target transcripts in the adult compound eyes and NORPA's well-documented role in phototransduction, the simplest interpretation of our observations would be that light-driven expression responses are mediated by visual phototransduction. Nevertheless, NORPA is known to be expressed outside of the visual organs [95], and it has been reported to affect functions unrelated to phototransduction, such as olfaction [96] and temperature-controlled clock gene oscillations [97]. Additionally, *norpA* loss-of-function mutants show a number of defects in circadian locomotor behavior. Their activity profiles reveal an advanced evening activity peak under LD conditions and a shortened intrinsic period length under DD conditions, and they are slow to adjust their behavior to shifting cycles of light and dark [98,99]. One possible interpretation of these observations is that NORPA plays a role in seasonal photoperiodic control of locomotor behavior. The *norpA* mutant phenotype partially mimics the effect of a shortened photoperiod, which also leads to advanced evening activity peaks and shortened period lengths [100,101]. Two recent studies [102,103] provide further evidence connecting *norpA* to seasonal control of daily locomotor activity patterns. Majercak et al. [103]and Collins et al. [102] reported that *norpA* mutants show abnormally high levels of splicing in the 3′ untranslated region of *per* mRNA. Increased splicing of *per* transcripts at this site had previously been shown to contribute to the advanced accumulation of PER protein and the advanced timing of evening locomotor activity that is observed for shorter photoperiods and lower temperatures [100]. Thus, NORPA's effect on splicing of *per* may be an important determinant of the “short day” locomotor behavior phenotype of norpA mutants. The sustained photic expression responses that we identify here may reflect yet another mechanism for flies to translate a seasonal environmental signal (photoperiod) into a set of molecular signals. Photoperiodic control of transcripts associated with functions in visual sensitivity *(trpl* and *CdsA)* and synaptic transmission *(Slob* and *dlg1)* may be relevant to adaptive responses in the visual system and the brain. NORPA's involvement in both regulating *per* splicing and mediating photoresponses at the transcript level raises questions as to if and how these two molecular functions are connected. One possibility is that both reflect NORPA-dependent selective regulation of mRNA stability that takes place in the compound eyes (and perhaps also the brain). Whether or not NORPA's function in circadian locomotor behavior involves some of the light-dependent expression responses that we have identified could be examined by targeted misexpression studies. The subset of transcripts that have been independently confirmed to exhibit both NORPA-dependent light responses and strong clock-dependent circadian regulation might be particularly relevant to these experiments.

### Conclusions

A new strategy for analyzing oscillatory patterns in microarrray data has allowed us to answer general questions about oscillatory gene systems in the fly head. By applying this strategy to 17 d of data, we were able to conclusively demonstrate that there are more than a hundred circadian transcript oscillations in the fly head. Additionally, in a search for rhythmic gene activity over a wide range of periods (from 12 to 48 h), we established that 24-h periodicity constitutes the only broad program of transcriptional oscillation. We further found that the *tim-*dependent clock is the sole transcriptional circadian clock in *Drosophila*. Thus, the fly appears to differ from cyanobacteria, protists, plants, and fungi, which are thought to possess multiple circadian clocks. Lastly, we found a novel, light-regulated system of gene regulation in *Drosophila* that is largely dependent on *norpA*-mediated phototransduction. This system regulates about the same number of genes as the clock, including a number of circadian genes. Our study defines three types of transcripts that oscillate in wild-type flies: those from purely clock-regulated genes, those that are purely photocycle-regulated, and those expressed by genes that respond to both inputs.

## Materials and Methods

### Fly strains.

This study made use of “wild-type” strains *y w* and *Canton S* as well as mutant strains *y w; tim^01^, y w per^0^, Clk^jrk^, cry^b^, norpA^39^, norpA^7^,* and *norpA^7^; tim^01^.*

### Microarray experiments.

After eclosion *y w* or *y w; tim^01^* were entrained to a 12-h:12-h LD cycle at 25 °C for 3 d and harvested onto dry ice at 4-h intervals during the next day of LD entrainment and an additional day of free-running in constant darkness (DD). Relative to Zeitgeber time 0 (ZT0) as the time of lights-on during the LD cycle and circadian time 0 (CT0) as the time corresponding to subjective lights-on during free run in DD, time courses were collected in a ZT2-ZT6-ZT10-ZT14-ZT18-ZT22-CT2-CT6-CT10-CT14-CT18-CT22 schedule. Heads were isolated by breaking up frozen flies and passing them through a set of sieves. RNA was prepared using guanidine-thiocyanate extraction followed by purification over a CsCl gradient. Additional purification of the RNA samples was achieved by applying them to Rneasy columns (Qiagen, Valencia, California, United States). Biotin-labeled cRNA probe was generated from 25 μg of purified RNA and hybridized as described previously [16,104].

Different combinations of newly collected and previously published microarray data were analyzed. All included experiments were obtained on Affymetrix Drosophila Genome 1 arrays and used a 1- or 2-d time-course format with 4-h sampling intervals The wild-type dataset for Figures 2A, 2B, 6, and 8 consists of four LD/DD time courses, one that is new (*y w* #3) and three that were described previously (*y w* #1, *y w* #2, *cn bw;* [16]). The comprehensive wild-type dataset used for Figures 2C and D is expanded to also include the published data from three other studies (three to five replicates for a 1-d DD time course, [18]; two replicates for 2-d LDLD and DDDD time courses [15]; 1-d wild-type time courses LD0, LD2, DD1, and DD2, [17]); A fifth study [19] could not be included in this analysis because raw data were not available. The wild-type control dataset in Figure 3A and 3B consists of time courses *y w #1* and #3, whereas the *tim^01^* time-course data used in Figures 3A, 3C, 6, and 8 consist of the two newly collected LD/DD time courses *y w; tim01* #1 and *y w; tim01* #2. The comparisons between wild-type and arrhythmic mutant LD and DD conditions in Figure 5 were made with equal amounts of wild-type and arrhythmic mutant data from two sources: our *y w #1, y w #3, *y w; tim01* #1, and y w; tim01* #2 datasets and the wild-type LD0, LD2, DD1, DD2 and *per^0^* mutant LD1, LD2, DD1, and DD2 data described in Lin et al. [17].

### Fourier analysis.

Microarray data for each experiment were prepared using the RMA signal algorithm [105]. Time-course experiments were appended and decomposed into Fourier modes as described in [104]. One additional adjustment was made for the Fourier analysis of the wild-type DD and wild-type LD + DD datasets used in Figures 2C, 2D, and 5. Two of the four 1-d DD time courses used here (DD1 and DD2; Lin et al. [17]) were collected in constant darkness on the third rather than the first day, raising the possibility that free-running period lengths somewhat shorter or longer than 24 h could cause apparent phase shifts in the data for these two days. Consistent with the original report on these data [17], analysis of the top-ranking circadian oscillators found apparent peak phases on the third day of DD to be abnormally advanced (unpublished data). In order to account for this effect, we applied a +2.5-h phase correction.

### Permutation null models and QQ plots.

We developed a method for estimating the prevalence of rhythmic transcript profiles in time-course microarray data relative to a permutation null model [104]. The null model was obtained by conducting 1,000 permutations of the time ordering within each experiment for all probe sets and calculating Fourier scores for the resulting fictitious time series. The 1,000 × (number of probe sets) fictitious Fourier scores were then divided into a number of quantiles equal to the number of probe sets represented in the original. The distributions of Fourier scores found in real data and the permuted data quantiles were then compared and illustrated in QQ plots.

The data for the QQ plots of Figures 2, 3, and 5 were prepared and standardized as in [16], so that a perfectly sine-shaped oscillation with any period is attributed a maximal score of 1. Prior to Fourier analysis, the data in Figures 2A, 2B, and 3 were mean-centered for each 2-d experiment, whereas the data in Figures 2C and 5 were separately mean-centered for each day. Only genes represented by probe sets with more than half of their data points above a 20th percentile signal threshold were considered.

### Spectral analysis.

The spectral analysis in Figure 2B was conducted on data from 8 d of wild-type data (4 × LD/DD): one set of newly collected LD/DD data and three that have been described previously [16]. For each probe set, the four mean-centered time traces (in RMA units, corresponding to the logarithm of expression levels) are fit to cosine functions 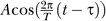 with periods *T* between 12 and 48 h and peak time τ. The analysis was done at three levels of stringency; sets were assembled from the number of transcripts with cosine fits with a residual variance (i.e., variance, not explained by the cosine fit) of less than 50%, 60% or 70% for each of the period lengths (12 h, 14 h, 16 h,…48 h).

### False discovery rates and background correction of transcript counts.

The *p*-value used in Figure 2D was obtained from a genome-wide permutation model and represents for each transcript profile the probability of observing a better or equal spectral power from a genome-wide set of randomly permuted profiles. The negative logarithm of this *p*-value is roughly proportional to the raw Fourier score. The benefit of using *p*-value cut-offs rather than Fourier cut-offs here is that they are less sensitive to the size of the dataset that is used and, therefore, allow for side-by-side comparisons of the four different datasets. FDR values associated with *p*-value cut-offs were calculated as the number of selected transcript profiles in randomly permuted control data divided by the number of selected transcript profiles in the real dataset. For each *p*-value cut-off, the background-corrected number of daily transcript oscillations (net counts) was obtained by subtracting the number of selected transcript profiles in randomly permuted control data from the number of selected transcript profiles in the real dataset (total counts). Possible contamination of LD/DD-selected circadian patterns with purely light-dependent profiles can be avoided by applying a filter based on 24-h spectral power in DD (available at http://flyfits.unil.ch).

### Selection of apparent *tim^01^* oscillators.

Three selections of microarray transcript profiles were used as the basis for a systematic survey of circadian oscillations in *tim^01^* flies. First, noisy transcript profiles were excluded by demanding that more than half of the 48 data points among the *tim^01^* (2× LD/DD) and wild-type control data (2× LD/DD: time course *y w* #1 and *y w* #3) be above the 20th percentile signal threshold. Second, we required that the absolute range for the four time courses on average exceed 0.5. Then, three selections were made based on Fourier analysis of RMA expression ratios for the data normalized per time course. The *pF_24_*-values associated with the 24-h Fourier scores of each probe set were calculated as in [16], using permutations of the real data within each experiment. The first selection simply ranks transcripts in ascending order according to the *pF_24_* value for the *tim^01^* data. The second selection uses the same ranking criteria, but excludes transcripts with a *pF_24_* for the wild-type data that exceeds 0.01. The third selection is ranked according to the *pF_24_* derived from joint analysis of the appended wild-type and *tim^01^* time courses. All three selections are limited to the 30 top-ranked transcripts.

### Selection of light-driven transcripts.

We considered the combined 6-d set formed by our wild-type (4 d) and *tim^01^* (2 d) LD time-course data, and required detection of reproducible daily oscillations in this context (*pF_24_* < 0.0075; calculated as in [16]). Additional selection steps were then taken based on datasets derived from the two *tim^01^* LD/DD time courses and two wild-type control LD/DD time courses (“*y w* #1” and “*y w* #3”). Two statistical tests were applied: A Mann-Whitney rank sum test comparing expression during the light phase (ZT2, ZT6, and ZT10) to that during the subjective light-phase (CT2, CT6, and CT10) was performed for the *tim^01^* data. In addition, a Kruskal-Wallis test was performed for the 4 d of combined wild-type and *tim^01^* LD data to determine if expression varies significantly with daily time during a photocycle (groups: ZT2, ZT6, ZT10, ZT14, ZT18, and ZT22). Transcripts were included if the probabilities for both tests did not exceed 0.1, and 0.05, respectively. The selection process was completed by the following three filters: First, we required that the absolute range values for wild-type LD and *tim^01^* LD on average exceed a threshold of 0.5. Second, we demanded that the observed average change in expression between day-time (ZT2, ZT6, and ZT10) and subjective day-time (CT2, CT6, and CT10) show the same direction in both wild-type and *tim^01^*. Third, to avoid profiles in which the light-driven component is of minor importance, we required that, for the *tim^01^* mutant, the variance observed during a light-dark cycle exceed the variance during constant darkness by more than 2-fold.

### Cluster analysis.

Microarray data corresponding to the final selection of 20 light-driven transcripts was used in a hierarchical cluster analysis with the CLUSTER and TREEVIEW software [106]. The normalized RMA expression ratios for each of four wild-type and two *tim^01^* time-course experiments were given equal weight in the analysis. Prior to clustering, transcript profiles were ordered by their estimated circadian peak phase across all of the wild-type LD data, as indicated in Figure 7A. The clustering solution shown in Figure 6 was obtained with the average linkage method using “uncentered correlation” as the similarity metric.

### Parametric model of light-driven and clock effects.

The RMA expression levels were fitted to the following model:


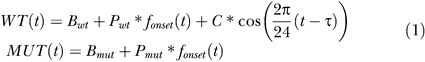


using standard least-squares regression on the combined wild-type and mutant data, totaling 10 d. Equal weight was given to all the data points; however, the data was not standardized here. The model assumes that the RMA profiles for wild-type *WT(t)* and *tim^01^* mutants *MUT(t)* are sums of a genotype-dependent baseline *B* and light susceptibility *P*. Only the wild-type data can have a circadian component of strength *C.* The peak phase is described by time τ, and the pulse function *f_onset_(t)* is a smoothed rectangular pulse of 12 h starting at time *onset,* taking value 1 when the lights are on and 0 otherwise. *onset* is the only parameter that is not fit, but determined by testing all integer values −2 to 6 and picking the best fit to the other six parameters. *onset* describes a delay of up to 6 h in the steady-state mRNA accumulation profile after the onset of lights, and is likely determined by the kinetics of accumulation and decay of each mRNA species. All fits can be visualized at http://flyfits.unil.ch. All parameters but τ have units of RMA ratio, so that the magnitude of the various contributions can be deduced from the reported values.

### Northern blot analysis.

For LD/DD time-course Northern analysis of various genotypes, except *norpA^7^* and *norpA^39^,* flies were harvested according to the same schedule used for microarray analysis (every 4 h during the fourth day of LD and an additional day of complete darkness). To minimize light-induced retinal degeneration in *norpA* mutant flies prior to harvesting, the flies were kept in the dark during development, a subsequent 4-d entrainment to a 12-h 25 °C/12-h 18 °C temperature cycle, and a 1-d free run at 25 °C. The samples for the *norpA^7^* and *norpA^39^* LD/DD time courses were then collected every 4 h during a single LD photocycle (in phase with the previous temperature cycles) and a subsequent day of free-run. Samples for Northern analysis of light-pulse–treated dark-reared *tim^01^* or *norpA^7^; tim^01^* flies were taken every 4 h for 1 d starting 2 h after the initiation of either a 6-h or 24-h light-pulse. Because the 6-h and 24-h light-pulse experiments sourced the same population of flies and were analyzed simultaneously on the same blots, their quantified results are directly comparable. RNA was prepared by guanidine-thiocyanate extraction followed by purification over a CsCl gradient and ethanol precipitation. Probe sequences were accessed from the Drosophila Genome Collection (Berkeley Drosophila Genome Project). A Storm Phosphorimager (Molecular Dynamics, Sunnyvale, California, United States) and IMAGEQUANT software (GE Healthcare, Piscataway, New Jersey, United States) were used for detection and quantitation of hybridized signals. Northern blots for ten genes revealed multiple hybridizing bands *(trpl, CG5798, Epac, CG2082, Slob, CG3799, dlg1, Pkc53E, CG31038,* and *CG30494)*. In most cases, the quantitative analyses are based on the band with the strongest hybridizing signal. The only exceptions are *Epac* and *Pkc53E,* for which additional hybridizing bands of approximately equal intensity were detected, and *CG5798,* for which an additional band of stronger intensity was found. After background subtraction, Northern signals were normalized to a loading control and graphed relative to the time-course average. Prior to Fourier analysis, Northern expression data was log_2_-transformed and normalized to the experimental average.

## Supporting Information

Figure S1 Northern Analysis of Putative Circadian Transcripts in *tim^01^* Fly Heads Survey on Northern blots confirms the absence of circadian transcript oscillations from fly heads mutant for *tim*. *y w; tim^01^* LD/DD time-course Northern profiles are shown for fourteen genes tested for circadian oscillations. After background subtraction, Northern signals are normalized to a loading control and graphed relative to the normalized time-course average (indicated as a horizontal line). Peak to trough expression ratios are indicated as P/T. Results from experimental repetitions are indicated for *CG8505*, *trpl*, *Pka-C3*, *CG5027*, *CG15211*, and *Inos*.
(88 KB PPT)

Figure S2 Examples of 48-h Oscillations Representing Light-Dependent Regulation Global analysis of the oscillatory statistics derived from our full wild-type dataset showed enrichment in 48-h gene expression rhythms (see Figure 2A). Such rhythms could indicate the presence of a novel, non-circadian oscillator active in wild-type fly heads. However, we favor another explanation, namely that the increase in 48-h periodic patterns reflects the presence of light-dependent gene responses. Recall that our experimental format used flies collected over a 48-h interval composed of 1 d of photo-entrainment (LD) followed by a second day of constant darkness (DD). We understand why such patterns appear as 48-h rhythms: An idealized time trace locked to the external light condition, namely a 12/36-h on/off step-like pattern, would lead to a spectral power of 0.55 at 48 h and 0.29 at 24 h. Note that 0.55 is close to the highest measured values found in the 48-h panel of Figure 2A, so a 12/36-h on/off pattern would be detected predominantly in the 48-h spectrum. Indeed, micorarray expression profiles with high *F_48_* scores reveal sustained responses to light. Three examples are shown. The graphs contain the normalized RMA expression values for LD/DD time courses using wild-type (four experiments; top panels) or *tim^01^* mutant flies (two experiments; bottom panels). The blocked horizontal bars below the cluster diagrams illustrate the environmental light-dark schedule used in the time courses, with white indicating light, black indicating darkness, and gray indicating subjective light under free-running conditions of constant darkness. The fitted lines were produced using a parametric model for circadian and light-dependent regulation (see Materials and Methods). Note the changes in relative expression corresponding to periods of light exposure in both wild-type and arrhythmic mutant *(tim^01^)* contexts.
(137 KB PPT)

Figure S3Northern Analysis of Light-Driven TranscriptsNorthern analyses of wild-type, *tim^01^, per^0^, Clk^jrk^, cry^b^, norpA^7^,* or *norpA^39^* LD/DD time-course data are graphed relative to the normalized time-course average (indicated as a horizontal line). Northern analyses are also shown for one-day time-course experiments in which a single 6-h or 24-h light pulse was given to dark-raised *tim^01^* or *norpA^7^ tim^01^* flies (see Materials and Methods). The 6-h and 24-h light-pulse profiles for each experiment are normalized to the average signal for the first two time-points (ZT2 and ZT6) in both profiles and shown respectively as black and gray lines. Peak to trough expression ratios for the LD part of LD/DD time courses or the 1-d light pulse experiments are indicated as P/T. P/T values below a noise threshold (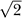) and P/T values for non–light-regulated patterns are indicated within parentheses and preceded by an X.(334 KB PPT)

Figure S4 Microarray Analysis of Light-Driven Transcripts Indicates Preferential Expression in the Compound Eyes RNA profiling analysis was performed for four replicate samples of adult head tissue derived from *eya^2^* mutant flies, which lack compound eyes, exposed to an environmental photocycle (see Protocol S1). RMA expression values were normalized to the average signal found for wild-type flies under the same conditions (average of LD time points from the four wild-type LD/DD time courses described in Figure 2A). The rows in the color-coded cluster diagram represent 27 light-driven genes (see Figure 7A) and the columns correspond to each of the replicates (columns 1–4) and the average (column “av”). The tree to the left represents the pairwise similarity relationships among genes according to their expression across the four replicates. The results of a Mann-Whitney rank sum test for differential expression in *eya^2^* versus wild-type heads are indicated in the column labeled *p*. Note that all light-driven genes show lower average expression in *eya^2^* and that in most cases, the expression differences are statistically significant. Light-dependent transcripts are preferentially down-regulated in *eya^2^* heads (prevalence of *eya^2^*-sensitive patterns [*pMW* < 0.01] is much higher in light-driven versus other expressed transcripts: Fisher's exact test *p* < 10^−12^).
A single asterisk (*), double asterisks (**), and triple asterisks (***) correspond to *p* < 0.05, *p* < 0.01, and *p* < 0.001, respectively.
(133 KB PPT)

Protocol S1 Supplemental Methods Microarray analysis of *eya^2^* adult heads was performed as described in Materials and Methods with the following modifications. *eya^2^* flies were collected onto dry ice at 6-h intervals during the fourth day of LD entrainment. Prior to RNA extraction, equal amounts of *eya^2^* heads were mixed for the four LD time points. One aliquot of mixed *eya^2^* heads was extracted as above, and a second aliquot was extracted with RNAzol (Tel-test, Iso-Tex Diagnostics, Friendswood, Texas, United States). cRNA was produced for the RNAzol-extracted sample in duplicate using either the Enzo BioArray Highyield ribonucleotide mixture (Enzo Life Sciences, Farmingdale, New York, United States) or a mixture of ribonucleotides with 6.25% representation each of bio-11-CTP and bio-16-UTP. cRNA for the guanidine-thiocyanate/CsCl–extracted aliquot was produced in triplicate using ribonucleotide mixtures containing either 8.3% representation of bio-11-CTP or 8.3% representation of bio-16-UTP or 6.25% representation each of bio-11-CTP and bio-16-UTP. The only obvious effect of varying the ribonucleotide mixture for cRNA production was a reduction in signal strength associated with the absence of bio-16-UTP (unpublished data). Data for the *eya^2^* cRNA sample lacking bio-16-UTP was, therefore, excluded from comparative quantitative analyses.
(24 KB DOC)

### Accession Numbers

The microarray data generated for this study has been deposited under accession number GSE3842 in the Gene Expression Omnibus data repository (http://www.ncbi.nlm.nih.gov/projects/geo). FlyBase (http://www.flybase.net) accession numbers for the *Drosophila* genes discussed in this paper are *14–3–3ζ* (FBgn0004907), *α-Man-IIb* (FBgn0026616), *CdsA* (FBgn0010350), *CG12120* (FBgn0030106), *CG14186* (FBgn0036935), *CG15211* (FBgn0030234), *CG1600* (FBgn0033188), *CG17352,* (FBgn0035880), *CG17386* (FBgn0033936), *CG2082* (FBgn0027608), *CG2121* (FBgn0033289), *CG30420* (FBgn0050420), *CG30494* (FBgn0027604), *CG31038* (FBgn0051038), *CG3799* (FBgn0027593), *CG4784* (FBgn0036619), *CG5027* (FBgn0036579), *CG5455* (FBgn0039430), *CG5798* (FBgn0038862), *CG7077* (FBgn0038946), *CG8468* (FBgn0033913), *CG8505* (FBgn0033728), *CG9317* (FBgn0032879), *CG9427* (FBgn0037721), *Clk* (FBgn0023076), *cry* (FBgn0025680), *dlg1* (FBgn0001624), *eag* (FBgn0000535), *Epac* (FBgn0033102), *eya* (FBgn0000320), *Inos* (FBgn0025885), *norpA* (FBgn0004625), *Pdf* (FBgn0023178), *Pdp1* (FBgn0016694), *per* (FBgn0003068), *Pka-C3* (FBgn0000489), *Pkc53E* (FBgn0003091), *rgr* (FBgn0033310), *Sh* (FBgn0003380), *slo* (FBgn0003429), *Slob* (FBgn0024290), *so* (FBgn0003460), *tim* (FBgn0014396), *to* (FBgn0039298), *trp* (FBgn0003861), *trpl* (FBgn0005614), *Tsp* (FBgn0031850), *Ugt35b* (FBgn0026314), and *vri* (FBgn0016076).

## Abbreviations

CT: circadian time
DD: dark/dark
FDR: false discovery rate
LD: light/dark
QQ: quantile–quantile
RMA: robust multi-array average
SCN: suprachiasmatic nucleus
ZT: Zeitgeber time
