# Supplementary material for: Control of Daily Transcript Oscillations in Drosophila by Light and the Circadian Clock
Source: PLoS Genet. 2006 Mar 24;2(3):e39. doi: 10.1371/journal.pgen.0020039 (PMC1413497; doi:10.1371/journal.pgen.0020039)
Supplement: Supplementary file 1 [file pgen.0020039.sd001.doc]

**Protocol S1.**

**Supplemental methods.**

Microarray analysis of *eya^2^* adult heads was performed as described in Materials and Methods with following modifications. *eya^2^* flies were collected onto dry ice at 6-hr intervals during the fourth day of light/dark entrainment. Prior to RNA extraction equal amounts of *eya^2^* heads were mixed for the 4 LD time points. One aliquot of mixed *eya^2^* heads was extracted as above and a second aliquot was extracted with Rnazol (Tel-test). cRNA was produced for the Rnazol-extracted sample in duplicate using either the ENZO bioarray Highyield ribonucleotide mixture or a mixture of ribonucleotides with 6.25% representation each of bio-11-CTP and bio-16-UTP. cRNA for the guanidine-thiocyanate/CsCl-extracted aliquot was produced in triplicate using ribonucleotide mixtures containing either 8.3% representation of bio-11-CTP or 8.3% representation of bio-16-UTP or 6.25% representation each of bio-11-CTP and bio-16-UTP. The only obvious effect of varying the ribonucleotide mixture for cRNA production is a reduction in signal strength associated with the absence of bio-16-UTP (data not shown). Data for the *eya^2^* cRNA sample lacking bio-16-UTP was, therefore, excluded from comparative quantitative analyses.
